# Naringenin exerts anticancer effects by inducing tumor cell death and inhibiting angiogenesis in malignant melanoma

**DOI:** 10.7150/ijms.44804

**Published:** 2020-10-18

**Authors:** Jawun Choi, Dae-Hyo Lee, Hyuk Jang, Sang-Youel Park, Jae-Won Seol

**Affiliations:** College of Veterinary Medicine, Jeonbuk National University, Iksan, Jeollabuk-do 54596, Republic of Korea

**Keywords:** Flavonoids, Naringenin, Apoptosis, Angiogenesis, Melanoma, HUVECs

## Abstract

Malignant melanoma is one of the most deadly skin cancer, due to its aggressive proliferation and metastasis. Naringenin, abundantly present in citrus fruits, has widely studied in cancer therapy. In this study, we investigated whether naringenin also has anticancer effects against B16F10 murine and SK-MEL-28 human melanoma cells. Moreover, we assessed the effects of naringenin treatment on angiogenesis of HUVECs and *ex vivo* sprouting of microvessels.Naringenin inhibited tumor cell proliferation and migration in a dose-dependent manner in B16F10 and SK-MEL-28 cells, which is supported by the results that phosphorylation of ERK1/2 and JNK MAPK decreased. Furthermore, naringenin induced cell apoptosis. Western blot analysisshowed naringenin treatment significantly upregulated the protein expression of activated cas3 and PARP in B16F10 and SK-MEL-28 cells. In addition, *in vitro* and *ex vivo* angiogenesis assays demonstrated that naringenin treatment potently suppressed EC migration, tube formation, and sprouting of microvessels. RT-PCR analysis showed that naringenin treatment significantly reduced the mRNA expression of Tie2, but did not inhibit the expression of Ang2. In conclusion, present study demonstrates the anticancer effects of naringenin by its induction of tumor cell death and inhibition of angiogenesis in malignant melanoma, suggesting that naringenin has potential as a safe and effective therapeutic agent to treat melanoma.

## Introduction

Malignant melanoma is the most lethal skin cancer, accounting for 60% of skin cancer-related deaths 1. The global incidence of melanoma has steadily continued to rise over the last two decades 2. Diagnosis of melanoma at an early stage followed typically by surgical excision is associated with a favorable prognosis. However, melanoma has a peculiar tendency to metastasize to distant organs at an early stage of progression. Thus, additional treatment, such as chemotherapy, radiation, and immunotherapy, is often required after surgery 3. In particular, although chemotherapy might be a suitable approach to treat metastatic melanoma, limitations of conventional chemotherapy include severe pain, adverse effects, and even recurrence 4. Therefore, the discovery of new compounds that are safe and effective against melanoma is becoming increasingly important.

Inhibition of tumor-associated angiogenesis has become a promising strategy for cancer therapy during the last decade. Angiogenesis, new blood vessel formation from existing vessels, plays an important role in various diseases including cancer. Sufficient blood vessel supply is essential for tumor growth and progression, since it provides oxygen and nutrients, and removes cellular metabolites. In addition, the tumor vasculature is the major route of metastasis of cancer cells from the primary site to distant organs. Anti-angiogenic effects of a variety of phytochemicals have been demonstrated in various cancer cells *in vitro* as well as using *in vivo* tumor models 5-7. The anti-angiogenic effect of gallic acid in ovarian cancer is due to the downregulation of the Phosphatase and tensin homologue deleted on chromosome 10/Protein kinase B/Hypoxia-inducible factor-1alpha (PTEN/AKT/HIF-1α) pathway and inhibition of vascular endothelial growth factor (VEGF) expression 8. The combination of curcumin and (-)-epigallocatechin-3-gallate (EGCG) has anti-angiogenic activities in colorectal carcinoma by blocking the Janus kinase/Signal transducer and activator of transcription 3/Interleukin-8 (JAK/STAT3/IL-8) pathway 9. Given the importance of angiogenesis in cancer, identification of plant-derived compounds with anti-angiogenic activity against cancer cells and minimal toxicity to normal cells is an important research goal.

Flavonoids derived from plants have been actively investigated for cancer chemotherapy because of their therapeutic effects against various human cancers. Naringenin is one of the most important plant flavonoids. It is abundantly present in citrus fruits that include grapefruit, orange, and mandarin. Previous studies have demonstrated extensive pharmacological properties for naringenin, which include antioxidant, anti-diabetic, and anti-inflammatory 10. Naringenin also has anticancer effects due to the induction of apoptosis and cell cycle arrest in a variety of cancer cell lines, including MDA-MB-231 breast tumor cells 11, HepG2 human hepatocellular carcinoma cells 12, E0771 mammary tumor cells 13, and PC3 and LNCaP prostate cancer cells 14. A recent study suggested an important role for naringenin as an anti-angiogenic chemopreventive agent 15.

The present study was undertaken to assess the simultaneous anticancer and anti-angiogenic bioactivities of naringenin. The findings suggest that naringenin can be developed as a safe and effective drug to treat melanoma.

## Materials and Methods

### Cell culture and reagents

B16F10 murine and SK-MEL-28 human melanoma cell lineswere purchased from the Korean Cell Line Bank (Seoul, Korea). The cells were cultured in Dulbecco's modified Eagle's medium (B16F10 cells) or modified Eagle's medium (SK-MEL-29 cells, Gibco, Grand Island, NY, USA) supplemented with 10% fetal bovine serum (Atlas Biologicals, For Collins, Co, USA), 100 U/ml penicillin and 100 μg streptomycin (Sigma-Aldrich). Human umbilical vein endothelial cells (HUVECs) and their growth medium (Endothelial Cell Growth Medium) were purchased from PromoCell GmbH (Heidelberg, Germany). The cells were used at passage 4-5 in all experiments. All cells were incubated at 37 °C with 5% CO_2_. Naringenin was dissolved in dimethyl sulfoxide to obtain a 200 μM stock solution, which was then diluted with media.

### Cell viability analysis

Cell viability was determined by a 3-(4,5-dimethylthiazol-2-yl)-2,5-diphenyltetrazolium bromide (MTT) assay. B16F10 and SK-MEL-28 cells were seeded in 24-well plates (1 × 10^5^ cells/well) overnight and treated with or without naringenin. After 24 h, the media was carefully removed and 300 μl of MTT solution (0.5 mg MTT/ml medium) was added to each well, and plates were incubated for 2 h at 37 °C. The media was then replaced with 500 μl of dimethyl sulfoxide, and the plates were shaken for 10 min. Then, 200 μl of the sample were transferred to a 96-well microplate. The results were quantified by measuring the absorbance at 570 nm with a microplate reader (Spectramax M2; Molecular Devices, CA, USA).

### Flow cytometry assays

Cell apoptosis was assessed by the Annexin V assay (Santa Cruz Biotechnology, Inc., Dallas, TX, USA) according to the manufacturer's protocol using a flow cytometry method. Annexin V content was determined by measuring fluorescence at 488 nm (excitation) and 525 nm (emission) using a Guava easyCyteHT system (Millipore, Billerica, MA, USA).

### *In vitro* scratch migration assays

Melanoma cell lines (B16F10 cells and SK-MEL-28 cells) and HUVECs were grown on 6-well plates. Confluent monolayer cells were scratched manually using a sterile 1000 μl pipette tip and gently washed with PBS. Fresh medium containing 1% fetal bovine serum was added to the wells with different concentrations of naringenin. At the indicated time after treatment, images were photographed using a microscope (Nikon Eclipse TS100; Nikon Corporation, Tokyo, Japan).

### *In vitro* tube formation assays

For endothelial cell (EC) tube formation assay, growth factor reduced MatrigelTM (Corning Inc., NY, USA) was thawed overnight at 4 °C. The Matrigel was allowed to solidify on a 24-well culture plate at 37 °C for 30 min. HUVECs were harvested and seeded at a density of 1 × 10^5^ cells/well in growth media with or without naringenin. Cells were then incubated at 37 °C for a further 18 h. Tube formation was observed by taking pictures using a microscope (Nikon Corporation). The tube formation assays were quantified by counting the number of tubules from three different fields for each condition.

### Rat aortic ring assays

For *ex-vivo* angiogenesis study, the rat aortic ring assay was used following a published protocol with modificiations^16^. Briefly, the thoracic aorta from a freshly sacrificed Sprague-Dawley rats (4-week-old, Samtako) was removed in a sterile manner and rinsed in ice cold PBS. It was then cut into 1 mm long pieces using surgical blade. Each ring was placed in a matrigel pre-coated 24-well-plate. Dulbecco's modified Eagle's medium containing 10% fetal bovine serum was added to the wells with or without naringenin. Seven days after treatment, the rings were analyzed by microscope (Nikon Corporation) and microvessels sprouting were quantified.

### RNA extraction and Reversetranscription (RT)-PCR

Total RNA was extracted using TRIzol® Reagent (Invitrogen, CA, USA) according to the manufacturer's instructions. The 2 µg of total RNA was reverse transcribed into cDNA using SuperScript II Reverse Transcriptase (Invitrogen). The cDNA aliquots were amplified on a Mycyler Thermal Cycler (Bio-Rad) using Go Tag DNA polymerase (Promega) and the gene primers listed in Table 1. Each PCR cycle consisted of 94 °C for 1 min, 60 °C for 1 min, and 72 °C for 1 min, The PCR products were loaded onto a 1.5% agarose gel containing LoadingSTAR nucleic acid dye (6X, Dynebio, Seongnam, Korea), electrophoresed, and photographed using a Fusion FX7 acquisition system (VilbertLourmat, Eberhardzell, Germany).

### Western blot analysis

At the indicated time, the cells were homogenized in ice-cold lysis buffer containing a protease inhibitor cocktail (Sigma-aldrich). Each protein was separated with SDS-PAGE and transferred to nitrocellulose membranes. After blocking with 5% skim milk, the membranes were incubated with the following primary antibodies in blocking buffer overnight at 4 °C: anti-caspase-3 (Cas3) (rabbit), anti-Poly (ADP-ribose) polymerase (PARP), phosphorylated extracellular signal-regulated kinases1/2 (p-ERK1/2) (rabbit), andphosphorylated c-Jnk N-terminal kinase (p-JNK) (mouse) (all from Cell Signaling Technology, Inc., Beverly, Ma, USA)and anti-β-actin (mouse monoclonal; Sigma-Aldrich). Membranes were then incubated with HRP-conjugated secondary antibodies for 1 h at RT. Chemiluminescent signals were developed with HRP substrate (Millipore) and detected with a Fusion FX7 acquisition system (VilbertLourmat, Eberhardzell, Germany).

### Immunocytochemistry

B16F10 cells and SK-MEL-2 cells cultured on glass coverslips coated with 0.1% gelatin. Cells were fixed with cold 2% paraformaldehyde and permeabilized with ice cold 0.5% Triton X-100 in PBS for 5 min and blocked in 5% donkey serum in 0.1% TritonX-100 in PBS for 1 h at RT. The cells were incubated with anti-active caspase-3 (rabbit polyclonal; R&D Systems) overnight at 4 °C. Cells were incubated with Cy3-conjugated donkey anti-rabbit IgG (Jackson ImmunoResearch). Nuclei were stained with 4'6-diamidino-2-phenylindole. Then, the cells were mounted in Fluorescent Mounting Medium (Dako) and immunofluorescent images were acquired using a confocal microscope (Carl Zeiss).

### Statistical analysis

Values are presented as the mean ± standard deviation (SD). Significant differences between groups were determined by unpaired Student t-tests. For multigroup analysis of variances, one-way or two-way ANOVA was performed followed by Bonferroni post-tests. All statistical analysis was performed using the GraphPad Prism software. Statistical significance was set at *p*< 0.05.

## Results

### Naringenin treatment inhibits viability and migration of B16F10 murine and SK-MEL-28 human melanoma cells

**Fig 1:**
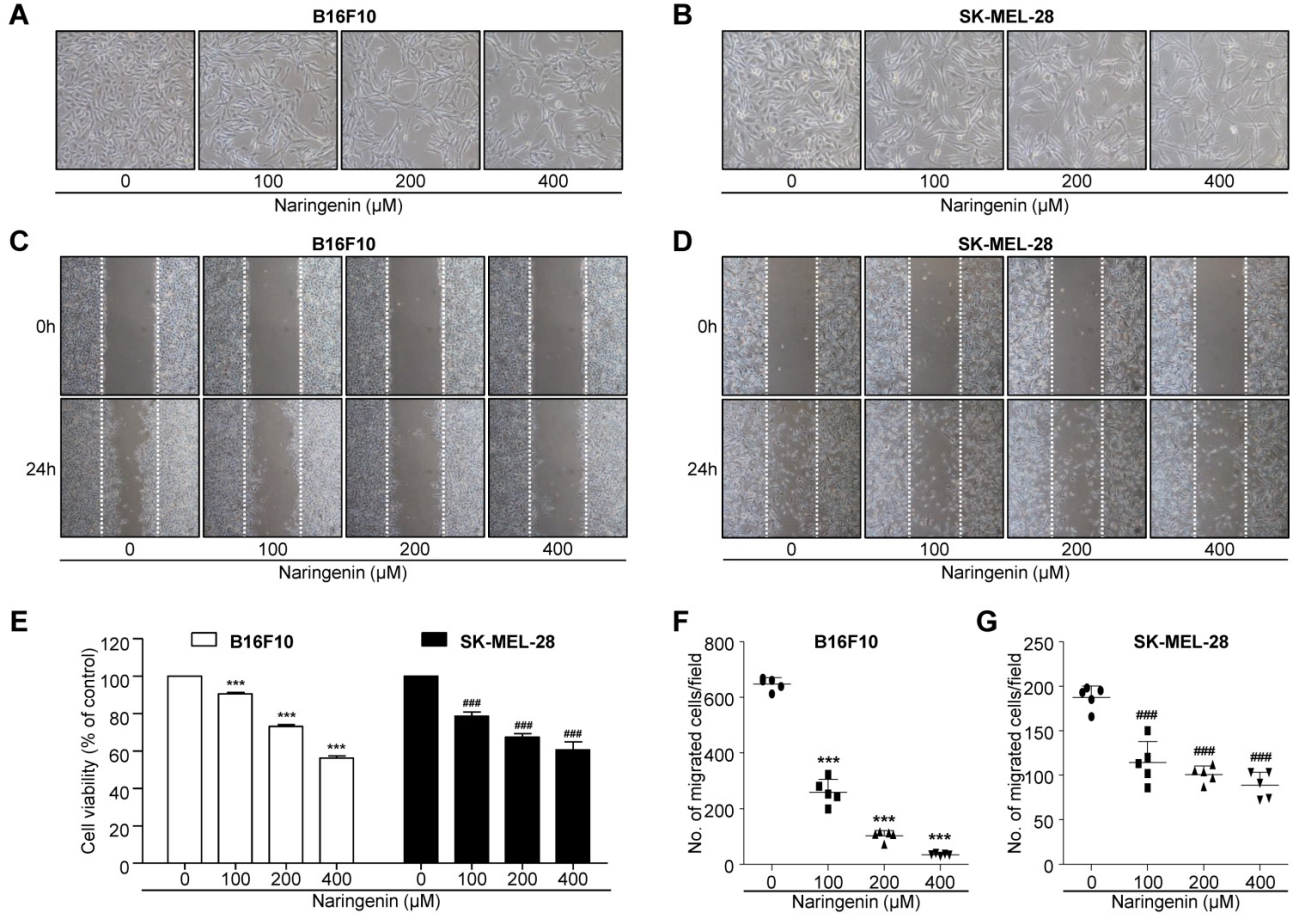
** Inhibitory effect of naringenin treatment on the tumor cell viability and migration. (A, B)** Images showing morphological changes on B16F10 murine (A) and SK-MEL-28 human (B) melanoma cells after naringenin treatment in a dose-dependent manner. Magnification, 100×.**(C, D)** Images showing B16F10 (C) and SK-MEL-28 (D) cell migration after naringenin treatment. Magnification, 40×. **(E)** Comparisons of B16F10 and SK-MEL-28 cell viability. **(F)** Quantification of B16F10 and SK-MEL-28 cell migration after naringenin treatment. Values are mean ± SD from ≥ three independent experiments. ***p< 0.0001; ###*p*< 0.0001 versus untreated cells by one-way ANOVA followed by Bonferroni post-tests.

B16F10 and SK-MEL-28 melanoma cells were treated for 24 h in the absence or presence of naringenin (100, 200, and 400 μM). Cell viability was determined by a standard MTT assay. The viability results are shown in Fig. 1A, 1B and 1E. Beginning at a concentration of 100 μM naringenin, B16F10 cell viability progressively decreased from 90.5% to 43.8% at 400 μM naringenin. Similarly, SK-MEL-28 cell viability was also suppressed with increasing concentration of naringenin, from 78.5% at 100 μM naringenin to 60.9% at 400 μM naringenin. The influence of naringenin on the migration of B16F10 and SK-MEL-28 melanoma cells was also assessed. Confluent cell monolayers were scratched and the subsequent migration of cells in the absence or presence of naringenin (100, 200, and 400 μM) was observed by optical microscopy. Naringenin at a concentration of 100, 200, and 400μM potently inhibited B16F10 cell migration by approximately 61.1%, 88.9%, and 96.7% versus untreated cells, respectively (Fig. 1C and 1F). Similarly, SK-MEL-28 cell migration was suppressed by 100, 200 and 400 μM naringenin by 38.3%, 43.8%, and 50.8% versus untreated cells, respectively (Fig. 1D and 1G).

### Effect of naringenin treatment on ERK1/2 and JNK signaling pathways

**Fig 2:**
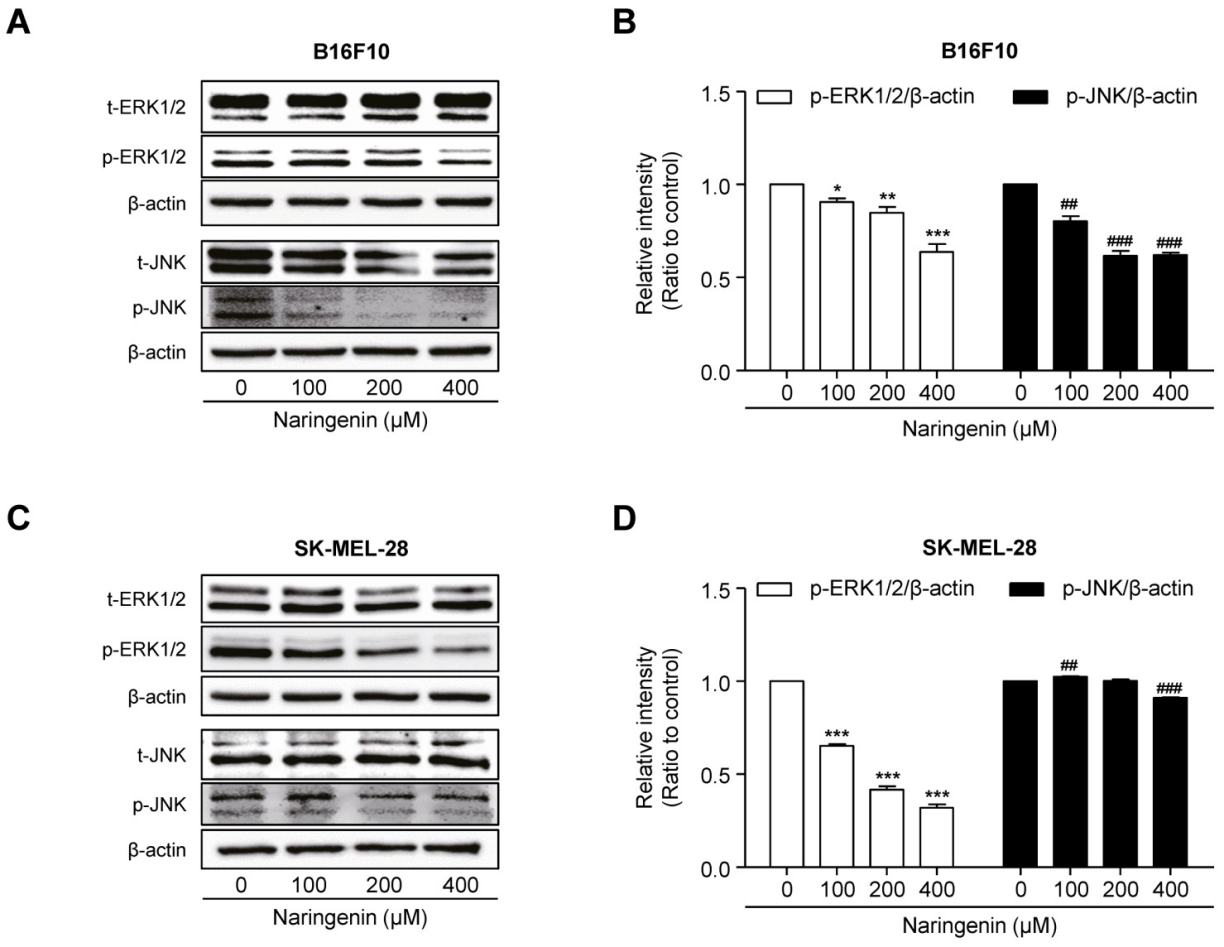
** Effect of naringenin treatment on ERK1/2 and JNK MAPKs signaling. (A-D)** Images and quantification of protein expression of p-ERK1/2 and p-JNK in B16F10 (A, B) and SK-MEL-28 cells (C, D) after naringenin treatment in a dose-dependent manner. Values are mean ± SD from ≥ three independent experiments. *p = 0.0265, **p< 0.0071, ***p= 0.0006; ##*p* = 0.0013, ###*p* = 0.0002 versus untreated cells by one-way ANOVA followed by Bonferroni post-tests.

To investigate the molecular mechanism by which naringenin inhibits melanoma cell viability and migration, B16F10 and SK-MEL-28 cells were cultured and then either not treated with 100, 200, and 400 μM naringenin or left untreated. After 18 h of treatment, in B16F10 cells, naringenin treatment suppressed the protein expression of not only p-ERK1/2 but also p-JNK in a dose-dependent manner as compared to that in untreated B16F10 cells (Fig. 2A and 2B).In SK-MEL-28 cells, naringenin treatment remarkably inhibited the expression of p-ERK1/2 protein in a dose-dependent manner whereas it slightly suppressed p-JNK expression (Fig. 2C and 2D).

### Effect of naringenin treatment on melanoma cell apoptosis

**Fig 3:**
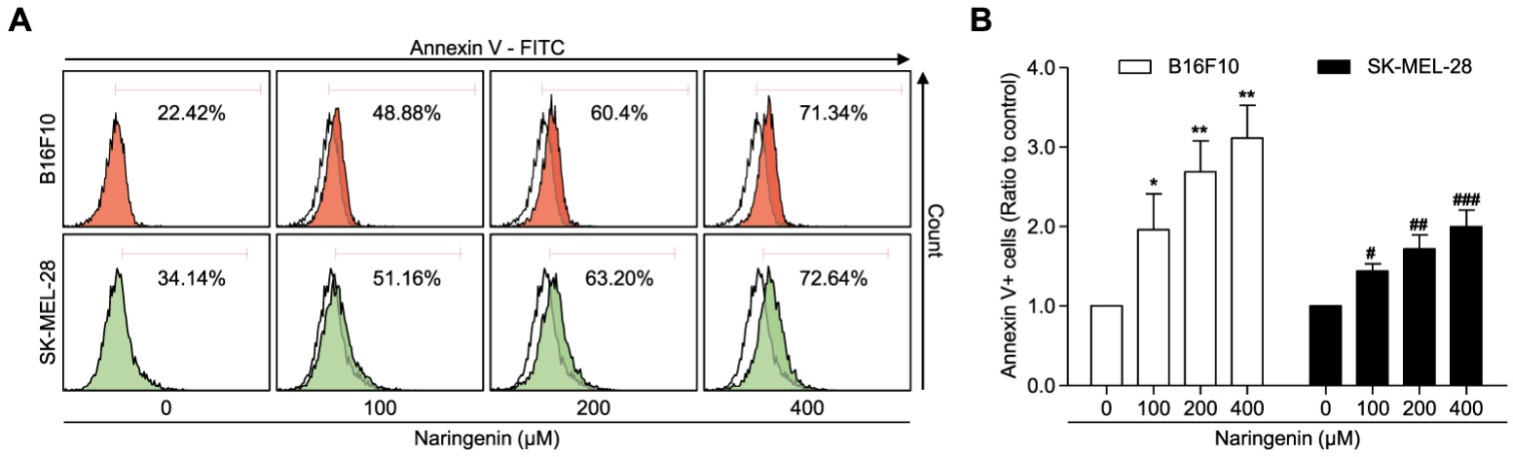
** Naringenin treatment induces cell apoptosis. (A, B)** Flow cytometry analysis and quantification showing apoptosis in B16F10 and SK-MEL-28 cells. Values are mean ± SD from ≥ three independent experiments. *p = 0.0248, **p< 0.006; #*p*< 0.0191, ##*p*< 0.004, ###*p*< 0.001 versus untreated cells by one-way ANOVA followed by Bonferroni post-tests.

To examine the effect of naringenin treatment on induction of cellular apoptosis, Annexin V/Propidium iodide double staining was used. Naringenin treatment induced apoptosis in the B16F10 and SK-MEL-28 melanoma cell lines in a dose-dependent manner (Fig. 3A and 3B). B16F10 cell apoptosis was markedly increased at 100, 200, and 400 μM naringenin more than from two to three times as compared to that of untreated cells. The apoptosis of SK-MEL-28 cells treated with naringenin was gradually increased to approximately two times at 400 μM as compared to that of untreated cells.

**Fig 4:**
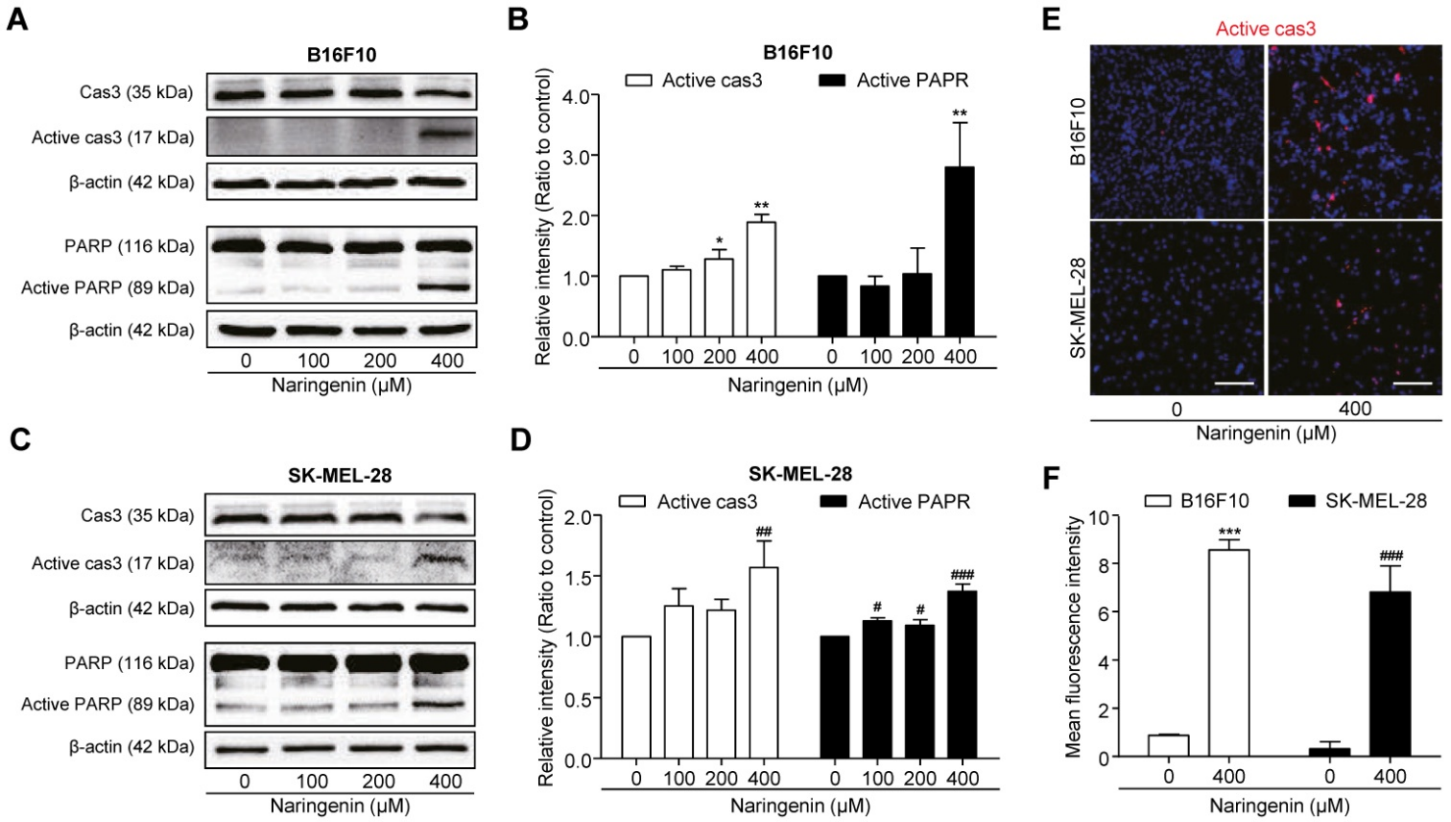
** Naringenin treatment leads to activation of cas3 and PARP. (A, B)**Images and quantification of active cas3^+^ area in B16F10 and SK-MEL-28 cells.Scale bar, 100 μm. **(C-F)**Images and quantification of protein expression of cas3, active cas3 and PARP, active PARP in B16F10 (C, D) and SK-MEL-28 cells (E, F) after naringenin treatment in a dose-dependent manner. Values are mean ± SD from ≥ three independent experiments. *p = 0.0340, **p = 0.0013, ***p < 0.0001; #*p*< 0.05, ##*p* = 0.01, ###*p*< 0.001 versus untreated cells by one-way ANOVA followed by Bonferroni post-tests.

To confirm the molecular signaling pathway associated with naringenin-induced apoptosis, we analyzed the expression of pro-apoptotic proteins by western blotting and immunocytochemistry in both the melanoma cell lines. Western blotting revealed the significant increase in activated cas3 level at 400 μM naringenin by approximately two times, as compared to that in untreated B16F10 cells (Fig. 4A and 4B). As PARP acts downstream to cas3, activation of cas3 by naringenin treatment induced the cleavage of PARP by approximately three times, as compared to that in untreated cells. Similarly, in SK-MEL-28 cells, 400 μM naringenin treatment significantly increased the intensity of active cas3 and active PARP signal by approximately one and a half times as compared to that in untreated cells (Fig. 4C and 4D).For Immunocytochemistry, we used a 400 μM concentration where induced cell apoptosis. Immunochemical data revealed naringenin activates the cas3 in B16F10 and SK-MEL-28, supporting above western blotting results (Fig. 4E and 4F).

### Naringenin exerts anti-angiogenic effects by inhibiting EC migration, tube formation, and microvessel sprouting

**Fig 5:**
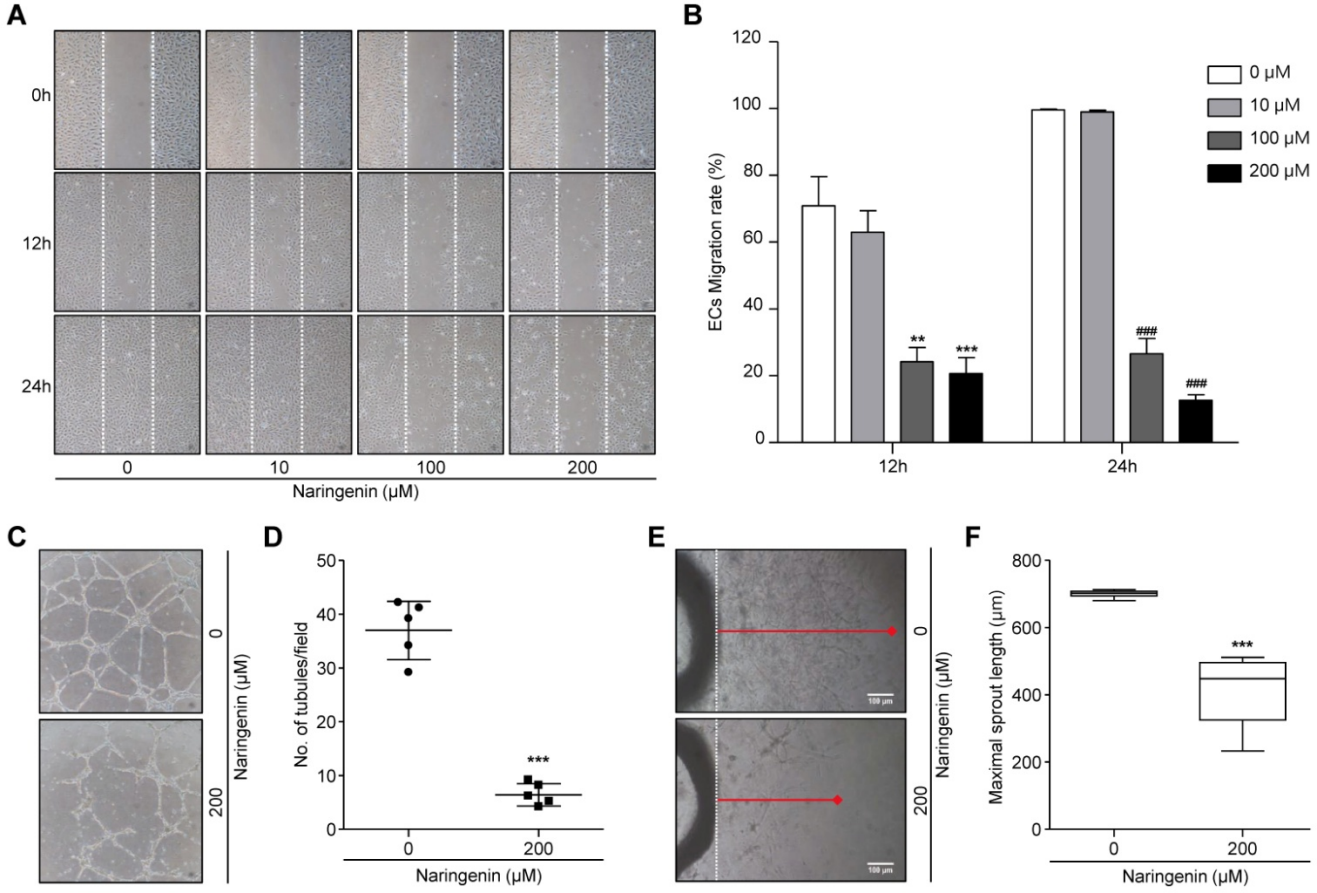
** Naringenin treatment inhibits angiogenesis *in vitro* and *ex vivo*. (A, B)** Images showing HUVECs migration and comparisons of ECs migration rate. Data are presented as a percentage of scratched area at time 0 h. Magnification, 40×. Values are mean ± SD from ≥ three independent experiments. **p = 0.0013, ***p= 0.0003; ###*p*< 0.001 versus untreated group by two-way ANOVA followed by Bonferroni post-tests. **(C, D)** Images showing ECs tube formation and comparisons of number of tubules. Magnification, 100×.**(E, F)** Images showing sprouting of ECs from rat aorta and comparisons of maximal length of ECs sprouting.Magnification, 40×. Values are mean ± SD from ≥ three independent experiments. ***p< 0.0001 versus untreated group by unpaired t-test.

To assess the inhibitory effects of naringenin on EC migration, HUVECs were seeded and allowed to grow to a confluent monolayer. The monolayer was scratched and then either untreated or treated with 10, 100, and 200 μM naringenin. After 12 h and 24 h, the cell migration was observed by optical microscopy. The representative photographs and their quantification showed the migration of HUVECs was markedly decreased following treatment with 100 and 200 μM naringenin (Fig. 5A and 5B). We used a 200 μM concentration for subsequent angiogenesis experiments. Since cell migration is crucial in tube formation of HUVECs 17, we suggest that naringenin may inhibit the tube formation of HUVECs as well as the cell migration. HUVECs were seeded on Matrigel with or without naringenin treatment and incubated for 18 h. The number of tubules was determined. Naringenin remarkably inhibited tube formation of HUVECs in a dose-dependent manner (Fig. 5C and 5D, Supplementary Fig. 1A and 1B). Furthermore, to assess the inhibitory effect of naringenin on microvessel sprouting, an *ex vivo* rat aorta ring assay was used. Treatment with 200 μM naringenin significantly attenuated the sprouting of microvessels from rat aorta ring in a dose-dependent manner (Fig. 5E and 5F, Supplementary Fig. 1C and 1D).

### Effects of naringenin treatment on expression of Ang2/Tie2

**Fig 6:**
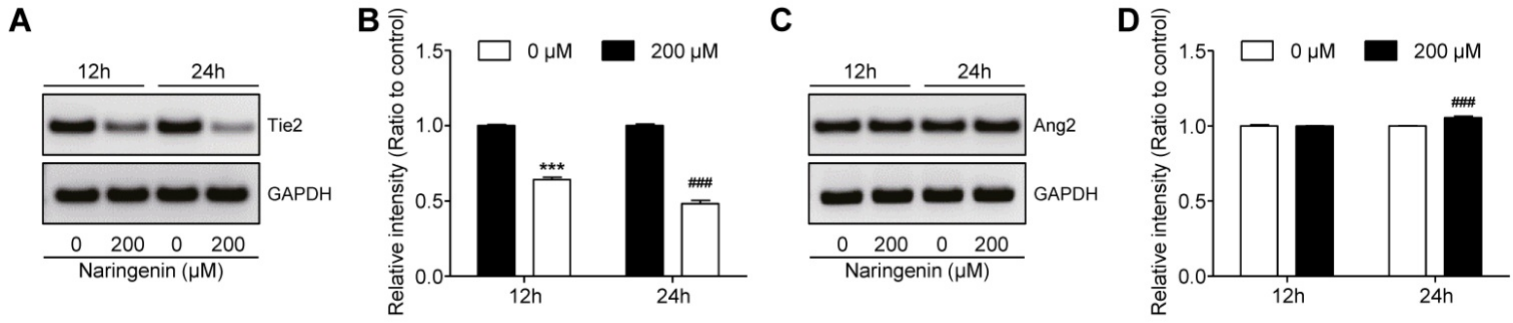
** Effect of naringenin treatment on expression of Ang2 and Tie2 mRNA in HUVECs. (A, B)** Images and quantification of mRNA expression Tie2 in HUVECs after naringenin treatment. **(C, D)** Images and quantification of mRNA expression Ang2 in HUVECs after naringenin treatment. Values are mean ± SD from ≥ three independent experiments. ***p< 0.0001, ###*p*< 0.0006 versus untreated group by unpaired t-test.

Since the Angiopoietin-2 (Ang2)/Tyrosine-protein kinase receptor-2 (Tie2) kinase signaling pathway is pivotal in tumor angiogenesis 18, we investigated the influence of naringenin treatment on this pathway. HUVECs were seeded and were either treated with 200 μM naringenin for 12 h and 24 h or left untreated. At each time, the mRNA expression of Ang2/Tie2 was confirmed by RT-PCR. Naringenin treatment remarkably reduced mRNA expression of the Tie2, as compared to that in untreated cells (Fig. 6A and 6B). However, naringenin treatment slightly affected the mRNA expression of Ang2 (Fig. 6C and 6D).

## Discussion

The documented therapeutic activities of naringenin, a major flavonoid, include anti-inflammatory 19, anti-diabetic 20, and anti-oxidative 21 activities. Particularly, naringenin exerts anticancer effects on various cancer cells 22-24. In the present study, we investigated whether naringenin also has anticancer effects against B16F10 murine and SK-MEL-28 human melanoma cells. We also assessed the effects of naringenin treatment on angiogenesis of HUVECs and *ex vivo* sprouting of microvessels.

Cell proliferation and migration of tumor cells are necessary processes during tumor progression. We observed that naringenin inhibited cell proliferation and migration in a dose-dependent manner in B16F10 and SK-MEL-28 cells. We further examined the phosphorylation status of Mitogen-activated protein kinases (MAPKs) by immunoblotting to elucidate the molecular mechanisms. MAPK signaling pathways, including ERK1/2 and JNK, are involved in survival and proliferation 25. Overexpression of phosphorylated MAPKs has been found in various cancer cell lines 26. In this study, we confirmed that naringenin suppressed the B16F10 and SK-MEL-28 cell growth by inhibiting the phosphorylation of ERK1/2 and JNK MAPKs. MAPK signaling pathway is initiated by activation of receptors including RTK in response to various extracellular signals such as growth factors, followed by activating a cascade signaling events of three kinases (i.e., MAPKKK-MAPKK-MAPK)^27^. Therefore, it may be suggested naringenin suppressed the activation of ERK1/2 and JNK MAPKs by targeting upstream proteins involved in the cascade of MAPK signaling.

The anti-cancer effect of naringenin on B16F10 melanoma cell line has been previously observed. Iwashita et al. revealed the growth inhibitory activity of naringenin on B16 mouse melanoma 4A5 cells 28. Bouzaiene et al. demonstrated that naringenin significantly suppressed the cell proliferation and induced the cell apoptosis on B16F10 cells 29. Similar to previous studies, we observed anti-proliferative activities of naringenin on B16F10 cells in a dose-dependent manner. However, our study differs from previous studies in two ways. First, we used human melanoma SK-MEL-28 cells. Second, we revealed the mechanisms underlying naringenin-induced cell apoptosis using protein expression analysis. In our studies, naringenin induced B16F10 and SK-MEL-28 cell death in a dose-dependent manner. Apoptosis is the most popular underlying mechanism of the anticancer effects of various anticancer drugs, including natural compounds 30.Similar to previous reports 31, 32, we found that naringenin killed tumor cells by inducing apoptosis. Furthermore, naringenin treatment significantly upregulated the expression of activated cas3 and PARP at 400 μM. Cas3 activate cleavage of PARP, and so is crucial in apoptosis. The present findings demonstrate that the increased activation of cas3 by naringenin treatment simultaneously induced PARP activation. These results indicate that naringenin induces apoptosis in a cas3-dependent manner.

Tumor angiogenesis is recognized as a pivotal cancer hallmark that is essential for tumor progression 33. Targeting tumor angiogenesis has been viewed as a promising strategy for clinical cancer therapy. However, clinical trials indicated that traditional anti-angiogenic treatment is limited and may instead accelerate tumorigenesis. In the present study, the results showed that naringenin treatment significantly reduced the migration of HUVECs in a dose-dependent manner. Moreover, the results of an *in vitro* EC tube formation assay on Matrigel and an *ex vivo* rat aorta ring assay demonstrated that naringenin treatment potently suppressed tube formation of HUVECs and sprouting of microvessels. Further study to understand the underlying mechanisms of the anti-angiogenic effects of naringenin, revealed that naringenin treatment significantly reduced the gene expression of Tie-2, but did not inhibit the expression of Ang2. These results suggest that the anti-angiogenesis effects of naringenin involve the downregulation of Tie2 expression.

Pafumi et al. demonstrated the anti-angiogenic effect of naringenin *in vitro* and *in vivo*^34^. Similar to our results, they showed that naringenin impaired not only VEGF-induced vessel formation but also neovascularization in C57BL/6 mice. However, our study is different from theirs in that we additionally investigated the inhibitory effect on HUVECs migration. Furthermore, to provide potential molecular mechanisms of the anti-angiogenic effects of naringenin, we identified the expression of angiogenic factors (i.e. Ang2, Tie2). However, they suggested that naringenin inhibits VEGF-induced angiogenesis by impaired intracellular calcium signaling.

**Fig 7:**
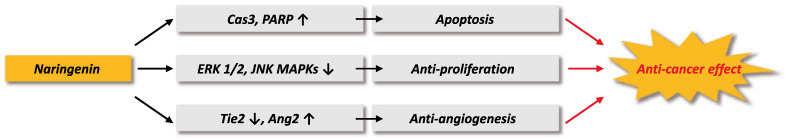
Schematic diagram of anti-cancer effect of naringenin.

In conclusion, we demonstrated the anticancer effect of naringenin and its potential mechanisms, as illustrated in Figure 7. Naringenin treatment suppressed proliferation and migration of B16F10 murine and SK-MEL-28 human melanoma cell lines by inhibiting the phosphorylation of ERK1/2 and JNK MAPKs. Naringenin treatment also induced cas3-dependent apoptosis in the melanoma cells, suppressed HUVECs migration and tube formation, and inhibited *ex vivo*microvessel sprouting by regulating the mRNA expression of Tie-2 and Ang-2. The collective results demonstrate the anticancer effects of naringenin via its induction of tumor cell death and inhibition of angiogenesis in malignant melanoma. Naringenin has potential as an effective and safe therapeutic agent for malignant melanoma.

## Supplementary Material

Supplementary figure.

## Figures and Tables

**Table 1:** Primer sequence used for RT-PCR.

Gene	Primer sequence	Size (bp)
Ang2	5'-GGATCTGGGGAGAGAGGAAC-3' 5'-CTCTGCACCGAGTCATCGTA-3'	535
Tie2	5'-ATCCCATTTGCAAAGCTTCTGGCTGGC-3' 5'-TGTGAAGCGTCTCACAGGTCCAGGATG-3'	512
GAPDH	5'-ACCACAGTCCATGCCATCAC-3' 5'-TCCACCACCCTGTTGCTGTA-3'	452
