# Supplementary figures and images for: Naringenin exerts anticancer effects by inducing tumor cell death and inhibiting angiogenesis in malignant melanoma
Source: Int J Med Sci. 2020 Oct 18;17(18):3049–57. doi: 10.7150/ijms.44804 (PMC7646117; doi:10.7150/ijms.44804)

Supplementary Figure 1.

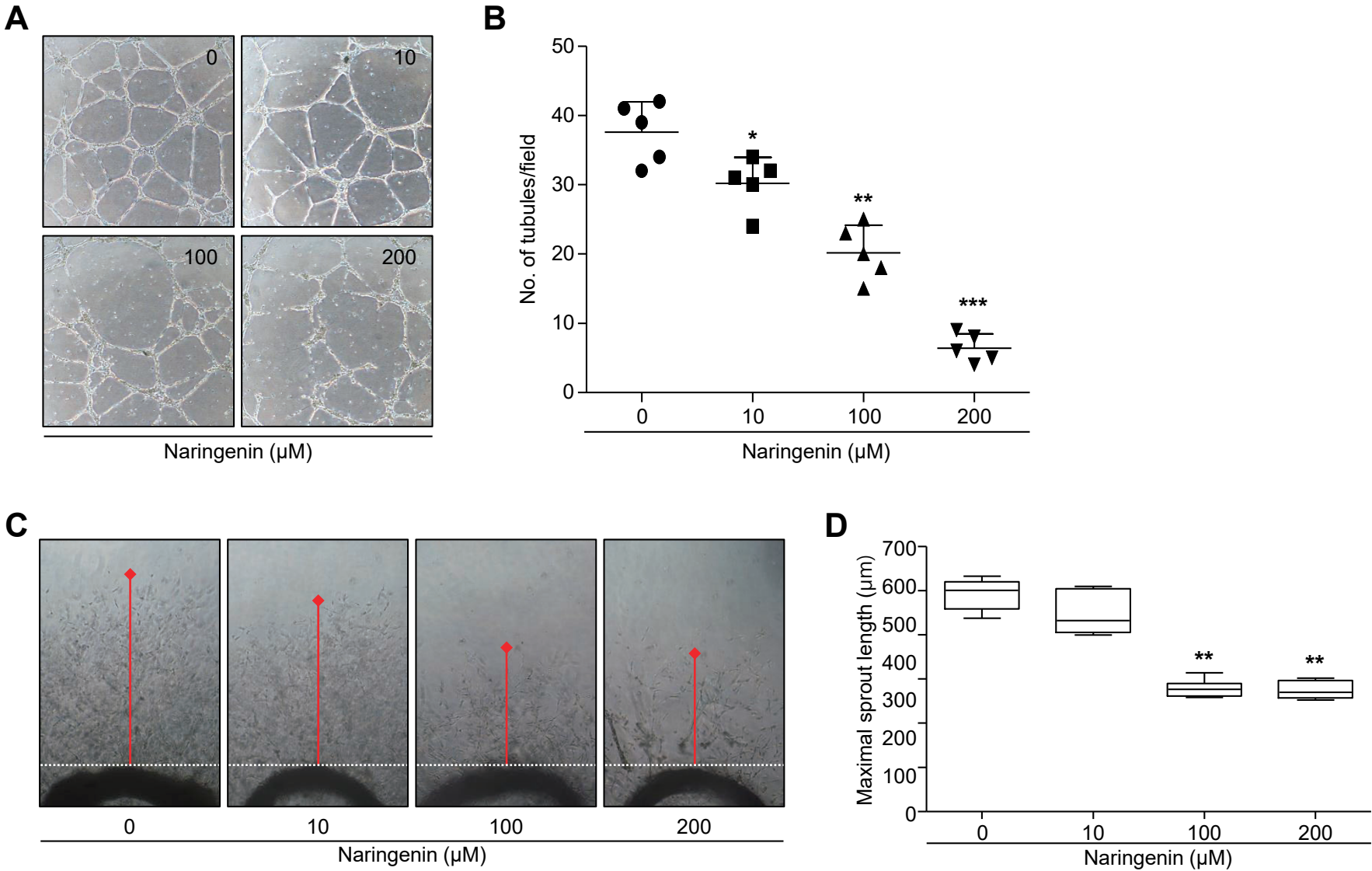

Supplement: Supplementary file 1 — Supplementary figure. [file ijmsv17p3049s1.pdf]
